# *Chlamydia psittaci* Pneumonia in a patient with motor neuron disease: a case report

**DOI:** 10.1186/s12879-023-08860-2

**Published:** 2023-12-05

**Authors:** Huade Luo, Lingling Jiang, Jie Chen, Dongying Wang, Yingying Kong, Guangli Cao

**Affiliations:** 1 Department of Emergency Intensive Care Unit, Shaoxing Second Hospital, Shaoxing, 312000 Zhejiang Province China; 2 Department of Blood donation service, Shaoxing blood center, Shaoxing, 312000 Zhejiang Province China

**Keywords:** Motor neuron Disease, *Chlamydia psittaci*, Bedside fiberoptic bronchoscope, Metagenomic next-generation sequencing, Severe Pneumonia, Case report

## Abstract

**Background:**

Motor neuron disease (MND) is a fatal neurodegenerative disorder that leads to progressive loss of motor neurons. *Chlamydia psittaci* (*C. psittaci*) is a rare etiology of community-acquired pneumonia characterized primarily by respiratory distress. We reported a case of *C. psittaci* pneumonia complicated with motor neuron disease (MND).

**Case presentation:**

A 74-year-old male was referred to the Shaoxing Second Hospital at January, 2022 complaining of fever and fatigue for 2 days. The patient was diagnosed of MND with flail arm syndrome 1 year ago. The metagenomic next-generation sequencing (mNGS) of sputum obtained through bedside fiberoptic bronchoscopy showed *C. psittaci* infection. Then doxycycline was administrated and bedside fiberoptic bronchoscopy was performed to assist with sputum excretion. Computed Tomography (CT) and fiberoptic bronchoscopy revealed a significant decrease in sputum production. On day 24 after admission, the patient was discharged with slight dyspnea, limited exercise tolerance. One month later after discharge, the patient reported normal respiratory function, and chest CT showed significant absorption of sputum.

**Conclusions:**

The mNGS combined with bedside fiberoptic bronchoscopy could timely detect *C. psittaci* infection. Bedside fiberoptic bronchoscopy along with antibiotic therapy may be effective for *C. psittaci* treatment.

## Background

Motor neuron disease (MND) is a fatal neurodegenerative disorder that leads to progressive loss of motor neurons, causing muscle weakness and wasting [1]. As respiratory muscles weaken, patients with MND experience breathing difficulties and are prone to respiratory complications and failure. In the late stage, ventilator support becomes necessary. Most patients with MND die from respiratory failure within 3 years of onset [2]. *Chlamydia psittaci* (*C. psittaci*) is an intracellular bacterial pathogen that causes parrot fever, a zoonotic disease that can cause severe pneumonia in humans [3]. Reports show that C. psittaci can be transmitted from person to person through a variety of ways [4]. *C. psittaci* has an incubation period ranging from 7 to 40 days, and its infection in humans presents with nonspecific symptoms such as fever, chills, coughing, shortness of breath, and chest pain [3]. Metagenomic next-generation sequencing (mNGS) is an effective method to identify rapidly and precisely a wide variety of pathogens [5]. Fiberoptic bronchoscopy is an examination that can be used bedside to obtain bronchoalveolar lavage fluid. However, their application in the diagnosis and treatment of *C. psittaci* lacks a consistent conclusion. Herein, we present a case of *C. psittaci* complicated with MND.

## Case presentation

**Fig. 1:**
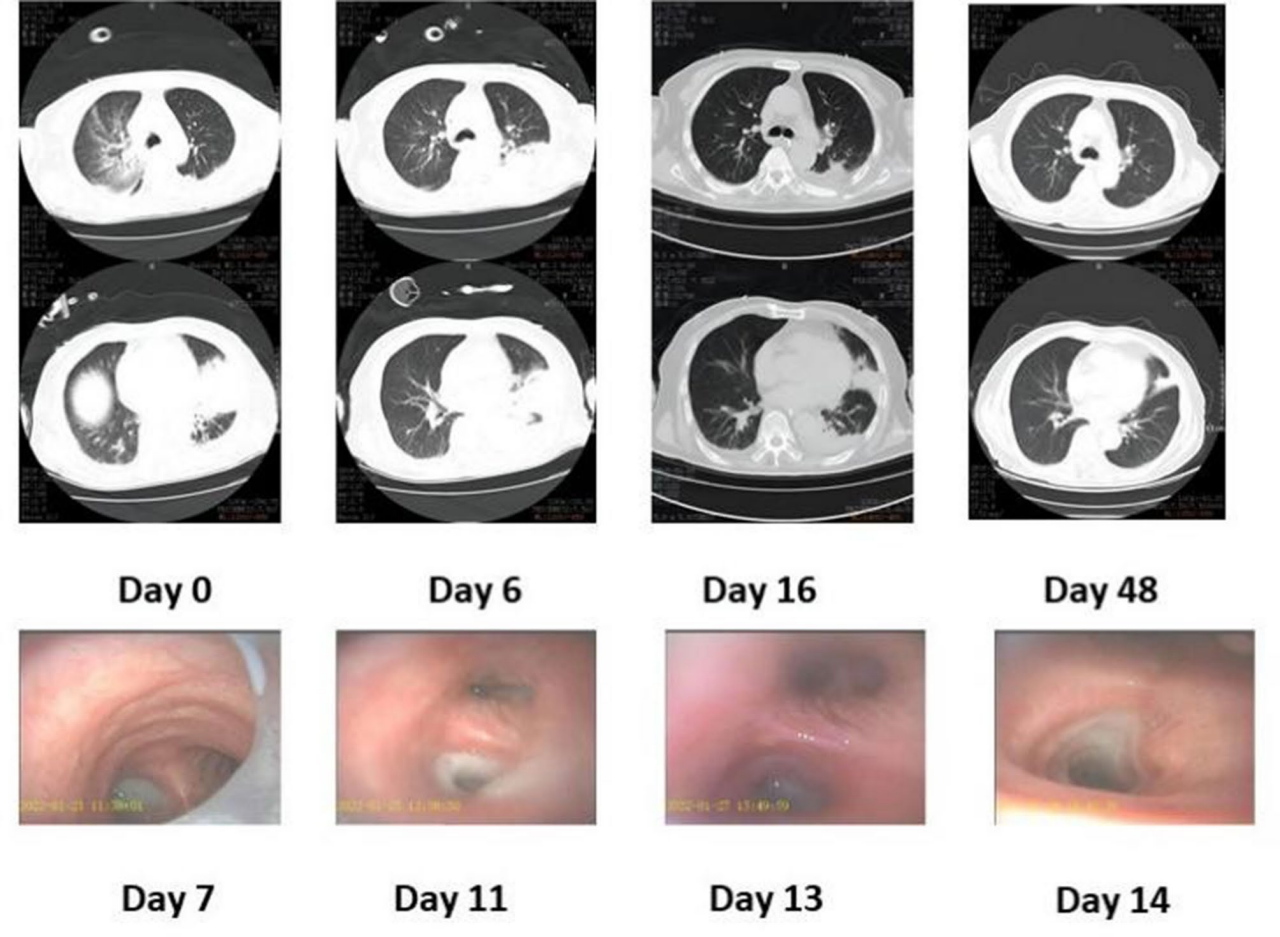
Results of fiberoptic bronchoscopy and chest CT. Fiberoptic bronchoscopy showed that trachea cannula and massive yellow purulent sputum in the left main bronchus on day 7. Reexamination on days 11, 13, and 14 showed a gradual decrease of sputum. The follow-up chest CT on the 6th day revealed an increased extent of pneumonia. After a 10-day course of doxycycline treatment and multiple bedside fiberoptic bronchoscopies with sputum aspiration, significant improvement in pneumonia was observed on the 16th, 22nd, and 48th days

**Fig. 2:**
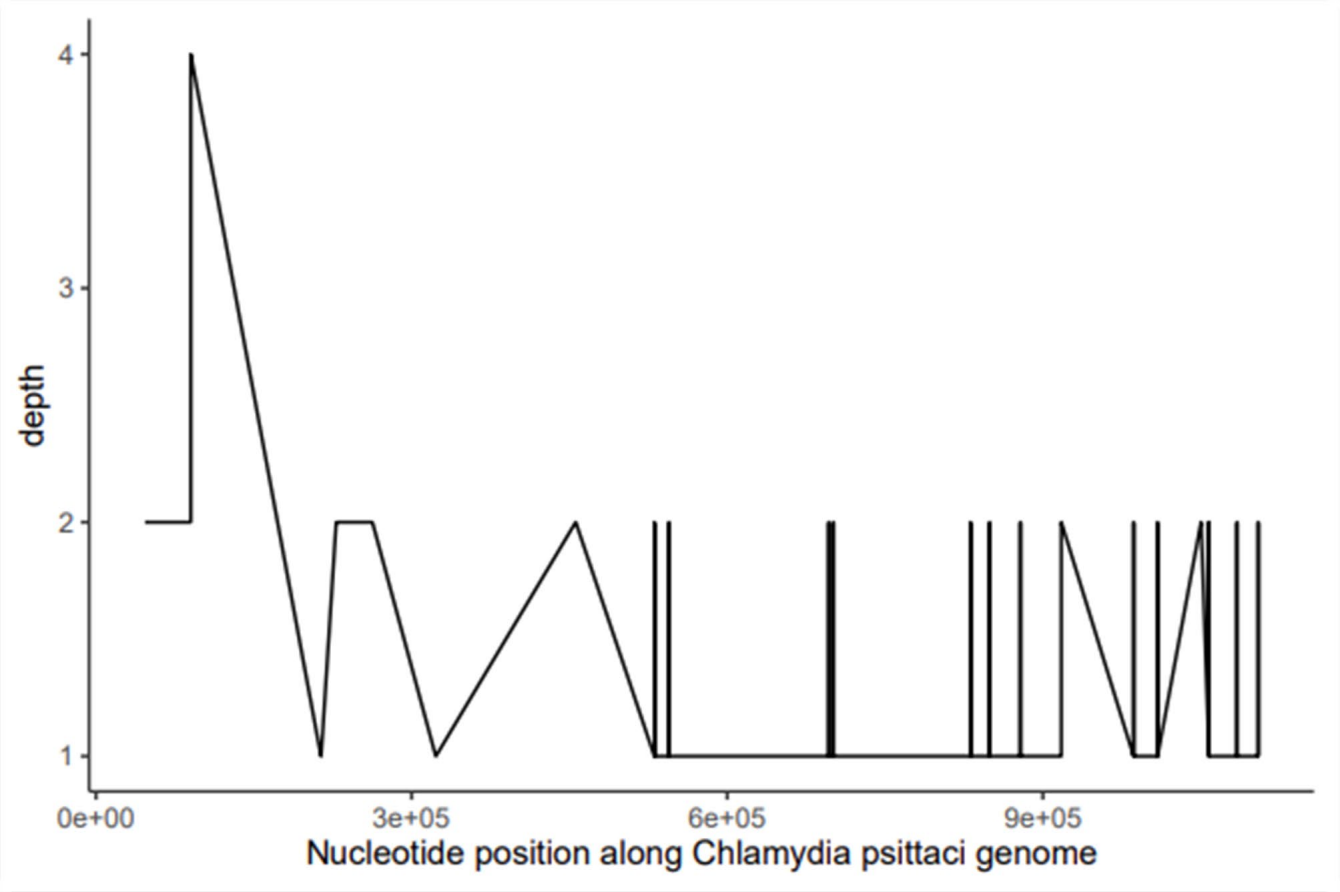
The result of mNGS of sputum suggested Chlamydia psittaci infection. X-axis represents nucleotide position along Chlamydia psittaci genome;Y-axis represents sequencing depth

A 74-year-old male was referred to the Shaoxing Second Hospital at January, 2022 complaining of fever and fatigue for 2 days. The patient was diagnosed with MND with flail arm syndrome (FAS) and severity grade stage II 1 year ago. Lung function tests were normal at that time. The patient was prescribed riluzole tablets 1 bid. so he resided in his hometown for convalescence, surrounded by lush trees and many birds living here. Since one month ago, the muscle strength of both upper limbs has further decreased and the patient was unable to raise his upper limbs above the shoulder. The family concurrently observed a reduction in the patient’s vocal intensity during speaking and coughing. No additional treatment has been administered. Physical examination showed that body temperature was 38.5 °C, pulse rate 131 beats/min, respiratory rate 22 beats/min, blood pressure 153/83 mmHg, breath sounds were coarse and lower in lung. The left thenar muscle, hypothenar muscle, and first interosseous muscle of both hands showed significant atrophy. Laboratory examination showed the white blood cell count was 12.7 × 10^9^/L, with an elevated neutrophil ratio of 93%. The concentration of C-reactive protein (CRP) was 243.4 mg/L. The concentrations of procalcitonin (PCT, normal < 0.05 ng/ml) and interleukin-6 (IL-6, normal < 5.4 pg/ml) were 11.5 ng/ml and 634.6 pg/ml. Arterial blood gas analysis showed a pH of 7.12, PaO_2_ of 76 mmHg, PaCO_2_ of 101.3 mmHg. Chest computed tomography (CT) showed pneumonia in the inferior lobe of the left lung (Fig. 1). Bedside fiberoptic bronchoscopy showed a large amount of yellow purulent sputum in the left main bronchus. Blood and sputum cultures, as well as other routine tests for pathogenic microorganisms, yielded negative results. The mNGS of sputum obtained through bedside fiberoptic bronchoscopy showed *C. psittaci* and elizabethkingia anophelis (Fig. 2). The patient was diagnosed as *C. psittaci* pneumonia complicated with MND.

The patient was administrated endotracheal intubation for mechanical ventilation. Imipenem and cilastatin sodium were administered for infection control initially, bromhexine hydrochloride was administered to decrease sputum production. Then doxycycline was administered after the diagnosis of *C. psittaci* pneumonia and bedside fiberoptic bronchoscopy was performed to assist with sputum excretion. After multiple suctioning, fiberoptic bronchoscopy revealed reduced sputum production (Fig. 1). The patient was successfully extubated on day 17 after admission. On day 24, he was discharged with slight dyspnea and limited exercise tolerance. One month later after discharge, the patient reported normal respiratory function, and chest CT showed significant absorption.

## Discussion and conclusions

This study reports a rare case of *C. psittaci* complicated with MND. This case highlighted the diagnostic and therapeutic value of mNGS and bedside fiberoptic bronchoscopy in the management of this case.

MND can cause respiratory muscle weakness, and when combined with psittacosis, it may lead to severe respiratory failure. The onset of the case was abrupt with high fever accompanied by fatigue, and rapid respiratory failure necessitating respiratory support. The patient may contract an infection through the inhalation of aerosols containing pathogens. *C. psittaci* pneumonia has atypical clinical manifestations and the diagnosis may be missed by traditional methods of microbiological diagnosis. The use of mNGS increases the probability of diagnosing *C. psittaci* [6]. The mNGS is highly sensitive for the detection of *C. psittaci*, the sequence reads that covered fragments of C. psittaci genome were detected more often in bronchoalveolar lavage fluid(BALF) than in blood samples [7]. Which can help clinicians identify the causative agent and guide clinical treatment. Tetracyclines, including doxycycline, are the preferred antibiotics for treating human psittacosis infection [8]. Bedside fiberoptic bronchoscopy is a non-invasive diagnostic and therapeutic tool that can provide the material for mNGS diagnosis and alleviate respiratory symptoms by removing excess sputum. Haowei T et al. found that the benefits of bedside fiberoptic bronchoscopy, in relieving respiratory symptoms, promoting lesion absorption, and improving clinical outcomes in patients with severe pneumonia [9]. There were several studies reported that bedside bronchoscopy combined with mNGS improved the diagnostic yield of pneumonia in critically ill patients compared with traditional culture methods [10, 11].

In conclusion, this case showed mNGS combined with bedside fiberoptic bronchoscopy may accurately detect *C. psittaci* infection. Bedside fiberoptic bronchoscopy along with antibiotic therapy may be effective for *C. psittaci* treatment.

## Data Availability

All data generated or analysed during this study are included in this article.

## Abbreviations

MND: Motor neuron disease
C. psittaci: Chlamydia psittaci
CT: Computed Tomography
FAS: Flail arm syndrome
