# Publisher Correction: Chlamydia psittaci Pneumonia in a patient with motor neuron disease: a case report

**DOI:** 10.1186/s12879-024-08990-1

**Published:** 2024-04-29

**Authors:** Huade Luo, Lingling Jiang, Jie Chen, Dongying Wang, Yingying Kong, Guangli Cao

**Affiliations:** 1 Department of Emergency Intensive Care Unit, Shaoxing Second Hospital, Zhejiang Province, Shaoxing, 312000 China; 2 Department of Blood Donation Service, Shaoxing Blood Center, Zhejiang Province, Shaoxing, 312000 China

**Publisher Correction: BMC Infectious Diseases 23, 852 (2023)**

**https://doi.org/10.1186/s12879-023-08860-2**

In the original publication of this article [1] figures 1 and 2 were incorrectly swapped. The captions and citations remain unchanged. The original article has been updated to rectify this error. The publisher apologizes to the authors & readers for the inconvenience.
